# Genetically predicted iron status was associated with the risk of prostate cancer

**DOI:** 10.3389/fonc.2022.959892

**Published:** 2022-12-06

**Authors:** Jiacheng Ying, Binyan Wang, Shuyang Han, Jie Song, Ke Liu, Weiwei Chen, Xiaohui Sun, Yingying Mao, Ding Ye

**Affiliations:** ^1^ The Fourth School of Clinical Medicine, Zhejiang Chinese Medical University, Hangzhou, China; ^2^ School of Public Health, Zhejiang Chinese Medical University, Hangzhou, China

**Keywords:** iron, ferritin, transferrin saturation, transferrin, prostate cancer, Mendelian randomization, meta-analysis

## Abstract

**Introduction:**

Observational studies have reported a relationship between iron status and the risk of prostate cancer. However, it remains uncertain whether the association is causal or due to confounding or reverse causality. To further clarify the underlying causal relationship, we conducted a Mendelian randomization (MR) analysis.

**Methods:**

We selected three genetic variants (rs1800562, rs1799945, and rs855791) closely correlated with four iron status biomarkers (serum iron, log-transformed ferritin, transferrin saturation, and transferrin) as instrumental variables. Summary statistics for prostate cancer were obtained from the Prostate Cancer Association Group to Investigate Cancer Associated Alterations in the Genome consortium including 79,148 cases and 61,106 controls of European ancestry. The inverse-variance weighted (IVW) method was conducted primarily to estimate the association of genetically predicted iron status and the risk of prostate cancer, supplemented with simple-median, weighted-median and maximum-likelihood methods as sensitivity analysis. MR-Egger regression was used to detect directional pleiotropy. We also conducted a meta-analysis of observational studies to assess the associations between iron status and the risk of prostate cancer.

**Results:**

Genetically predicted increased iron status was associated with the decreased risk of prostate cancer, with odds ratio of 0.91 [95% confidence interval (CI): 0.84, 0.99; *P* = 0.035] for serum iron, 0.81 (95% CI: 0.65, 1.00; *P* = 0.046) for log- transformed ferritin, 0.94 (95% CI: 0.88, 0.99; *P* = 0.029) for transferrin saturation, and 1.15 (95% CI: 0.98, 1.35; *P* = 0.084) for transferrin (with higher transferrin levels representing lower systemic iron status), using the inverse-variance weighted method. Sensitivity analyses produced consistent associations, and MR-Egger regression indicated no potential pleiotropy. Our replication analysis based on FinnGen research project showed compatible results with our main analysis. Results from our meta-analysis similarly showed that serum ferritin [standardized mean difference (SMD): −1.25; 95% CI: −2.34, −0.16; *P* = 0.024] and transferrin saturation (SMD: −1.19; 95% CI: −2.34, −0.05; *P* = 0.042) were lower in patients with prostate cancer compared with that in controls.

**Discussion:**

Our study suggests a protective role of iron in the risk of prostate cancer, further investigations are required to clarify the underlying mechanisms.

## 1 Introduction

Prostate cancer is the second most frequently diagnosed cancer and the fifth leading cause of cancer death in men, with 1.4 million new cases and 375,000 deaths worldwide in 2020 (1). Because aggressive forms of prostate cancer could commonly metastasize to bone and other parts of the body (2–4), the overall survival rate in cases with advanced and metastatic prostate cancer decreases markedly than those with localized prostate cancer, ranging from 26% to 30% at 5 years (5). To date, well-established risk factors of prostate cancer included advanced age, ethnicity, family history, and certain genetic mutations (1), whereas other risk factors remain undefined, such as nutritional status.

Iron is an essential element in basic biological processes and tightly linked to the utilization of oxygen and DNA synthesis (6). Accumulating observational studies have explored the associations of serum iron biomarkers (iron, ferritin, transferrin saturation, and transferrin) with the risk of prostate cancer. However, findings are conflicting. For example, a case-control study in America including 34 prostate cancer cases and 84 controls suggested that serum iron, ferritin, and transferrin saturation levels were lower in patients with prostate cancer than that in controls (7). However, a cohort study based on 114,847 Swedish populations including 1,505 patients with prostate cancer did not find statistically significant associations between serum iron and risk of prostate cancer (8). The inconsistent results from these studies may be due to differences in the study population, sample size, study design, and so on. In addition, the associations derived from conventional observational studies are susceptible to reverse causation and residual confounding (9). Therefore, it remains unclear whether the observed association was causal or not.

Mendelian randomization (MR) is a type of instrumental variable (IV) analysis for causal inference by utilizing genetic variants, such as single-nucleotide polymorphisms (SNPs) (10). SNPs are assumed to be randomly distributed in the general population in terms of Mendel’s laws of inheritance, which simulates a randomization process and thus enables MR analysis to minimize the confounding bias (11). As the genotype is randomly allocated at conception and cannot be changed by disease status, reverse causality could be eliminated in MR analysis. Moreover, a two-sample MR study design is harnessed for greater statistical power by obtaining genetic data in different populations (12).

In the current study, we conducted a two-sample MR study to assess the associations between iron biomarkers and the risk of prostate cancer. We also conducted a meta-analysis of observational studies to orient on the associations between iron status and the risk of prostate cancer.

## 2 Materials and methods

### 2.1 Mendelian randomization

**Figure 1:**
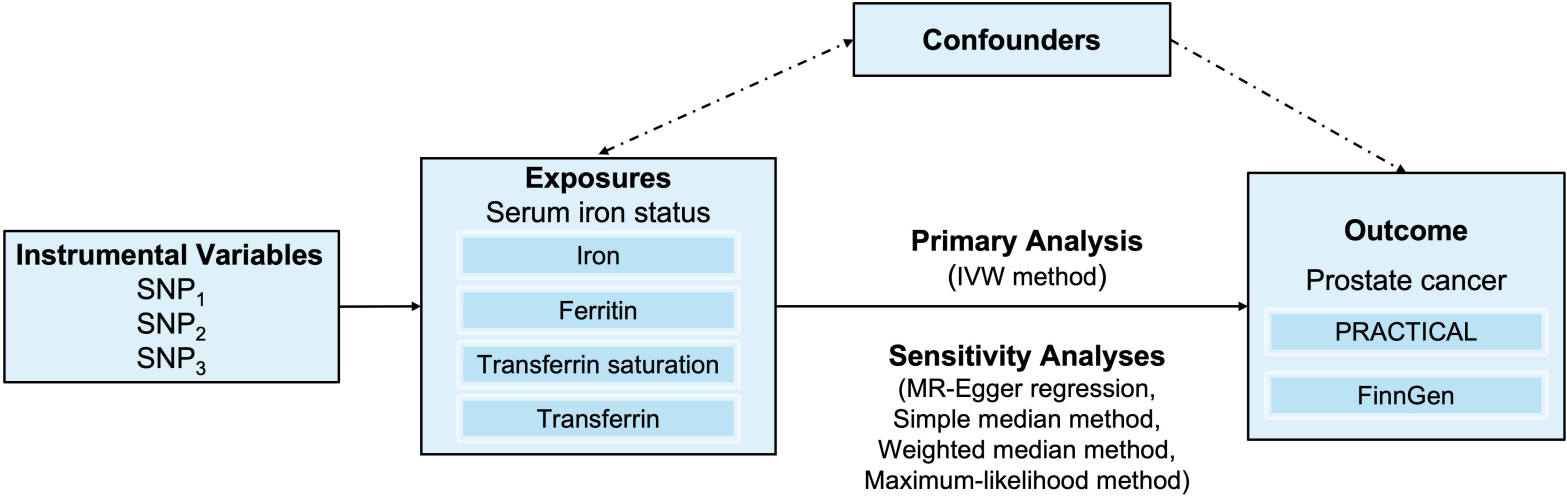
An overview of the study design. Abbreviations: IVW, inverse-variance weighted; SNP, single-nucleotide polymorphism. PRACTICAL, Prostate Cancer Association Group to Investigate Cancer Associated Alterations in the Genome.

The overall design for the current study is shown in **Figure 1**. We conducted a two-sample MR study to assess the associations between iron status (respectively reflecting serum iron, log-transformed ferritin, transferrin saturation, and transferrin) and the risk of prostate cancer based on summary data from genome-wide association studies (GWASs).

#### 2.1.1 Data sources

Summary statistics of prostate cancer in main analysis were obtained from a GWAS meta-analysis involving 79,148 cases and 61,106 controls of European ancestry conducted by Prostate Cancer Association Group to Investigate Cancer Associated Alterations in the Genome (PRACTICAL) consortium (13). The study performed a meta-analysis combining the summary statistics from a custom high-density array-OncoArray and seven previous prostate cancer GWAS or high-density SNP panels imputed to the 1000 Genomes Project of European ancestry. The summary statistics for replication were obtained from FinnGen database, containing 10,414 prostate cancer cases and 124,994 controls (14). The detailed information of the two projects (PRACTICAL and FinnGen) is listed in **Supplementary Table 2**. Because summary statistics of published studies were used, no additional ethical approval from an institutional review board was required.

#### 2.1.3 Selection of instrumental SNPs

Instrumental SNPs of iron status were derived from a meta-analysis of 19 GWASs performed by the Genetics of Iron Status Consortium with a total sample size of 48,972 European individuals (15). Three SNPs (rs1800562, rs1799945, and rs855791) tightly related to four iron status biomarkers (all *P*-values< 5 × 10^−8^ and pairwise *r* ^2^< 0.01) and consistent with an effect on systemic iron status (i.e., concordantly associated with increased levels of serum iron, log-transformed ferritin and transferrin saturation, and decreased levels of transferrin) were selected as IVs (**Supplementary Table 1**). The details of GWAS studies and datasets used in the present study are listed in **Supplementary Table 2**.

We calculated the proportion of variance (R^2^) explained by SNPs, using the formula 2×*MAF*×(1−*MAF*)×*β* ^2^ (16). F-statistic was performed to assess the strength of the instrument (17). The minimum detectable odds ratios (ORs) to provide 80% statistical power at a significance level of 5% were calculated using an online tool (https://shiny.cnsgenomics.com/mRnd/) (18).

#### 2.1.4 Statistical analysis

We first used the inverse-variance weighted (IVW) method to estimate the potential causal associations between genetically predicted iron status and the risk of prostate cancer. The IVW method combines the causal effect estimates of each IV by Wald ratio, having the greatest statistical power when all IVs are valid or the average pleiotropic is zero (balanced pleiotropy) (19, 20). A random-effects model was used to pool the Wald estimates in consideration of heterogeneity between SNPs. Cochran’s Q test was used to assess the heterogeneity from IVs due to pleiotropy or other causes. We further conducted several sensitivity analyses to evaluate the robustness of the results from IVW method. The simple median estimator can be thought of as a weighted median estimator with equal weights, which provides a consistent estimate of causal effect if at least 50% of IVs are valid, and the weighted median provides a robust estimate if at least 50% of the weight comes from valid IVs (21). Moreover, we performed MR-Egger regression to detect violations of the IV assumptions and assess potential directional pleiotropy. The intercept term was calculated as part of the analysis in MR-Egger method and could be regarded as the average pleiotropic effect across the genetic variants. The test on whether the intercept term differs from zero is so-called the MR-Egger intercept test (22).

All statistical analysis were performed using the “Mendelian Randomization” package in R version 4.1.0. *P*-values less than 0.05 were considered statistically significant, unless otherwise noted.

### 2.2 Meta-analysis

#### 2.2.1 Search strategy

We conducted a systematic search through PubMed and Web of Science for publications on the associations between iron status and prostate cancer until 12 March 2022. The review protocol of this meta-analysis was registered in PROSPERO (CRD42022316784). The search terms used were as follows: (iron OR Fe OR ferritin OR hyperferritinemia OR transferrin OR transferrin saturation) AND (prostate cancer OR prostatic cancer OR prostate neoplasms OR prostatic neoplasms).

#### 2.2.2 Literature inclusion and exclusion criteria

The inclusion criteria were as follows (1): study design of cross-sectional, case-control, or cohort; (2) prostate cancer cases and controls were distinguished according to clear diagnostic definition; and (3) the effect size of the association between iron status and prostate cancer was available, or there were sufficient data to calculate. The exclusion criteria were as follows: (1) article type of conference paper, protocol, abstract, review, or meta-analysis; (2) duplicate studies from different databases; and (3) if the population of individual studies overlapped, then the study with a smaller sample size was excluded.

#### 2.2.3 Data extraction and quality assessment

Two authors (Song Jie and Ying Jiacheng) separately extracted data from each eligible study onto a form with standard specifications, including first author, publication year, region, type of study design, sample size, and effect size. All the extracted information was checked, and the differences were solved by discussion. Quality assessments were conducted according to the modified Newcastle-Ottawa Scale (NOS) with scores ranging from 0 to 9 points (23). Studies with a quality score of 7–9 were regarded as high quality, 5–6 as moderate quality, and ≤4 as low quality.

#### 2.2.4 Statistical analysis

The mean and standard deviations of iron status and sample sizes between patients with prostate cancer and controls were used to calculate standardized mean differences (SMDs) and 95% confidence intervals (CIs) (24). For studies that provided OR, we converted OR to SMD by the formulae of $SMD=\left(\frac{\sqrt{3}}{\pi }\right)\times \ln OR$ (25) , and relative risk (RR) and hazard ratio (HR) approximate OR because the incidence of prostate cancer is relatively low (26). If the exposure category (iron status) was equal or greater than 3, then we used the lowest category as the reference and combined the risk estimates into a single estimate.

Cochrane’s Q test and *I* ^2^ value were used to assess the heterogeneity (*I* ^2^< 50% and *P* > 0.10, a fixed-effects model was used; otherwise, a random-effects model was applied). *I²* values of 25%, 50%, and 75% were considered low, moderate, and high heterogeneity, respectively. Publication bias was assessed by Egger’s test (27) and Begg’s test (28).

All statistical analyses were performed using STATA (version 15.1). Two-sided *P*-values less than 0.05 were considered statistically significant, unless otherwise noted.

## 3 Results

### 3.1 MR analysis

The F statistics for the instrumental SNPs ranged from 46.90 to 2,126.74 across the four biomarkers of iron status, all of which reached the conventional threshold of 10 (known as a rule of thumb to identify strong or weak instruments). The proportion of variance explained by the selected instrumental SNPs was about 3.9%, 0.8%, 7.3%, and 3.3% for serum iron, log-transformed ferritin, transferrin saturation, and transferrin, respectively. The minimum detectable ORs were 1.08, 1.19, 1.06, and 1.09, respectively, with 80% statistical power. Detailed information is listed in **Supplementary Table 3**.

**Figure 2:**
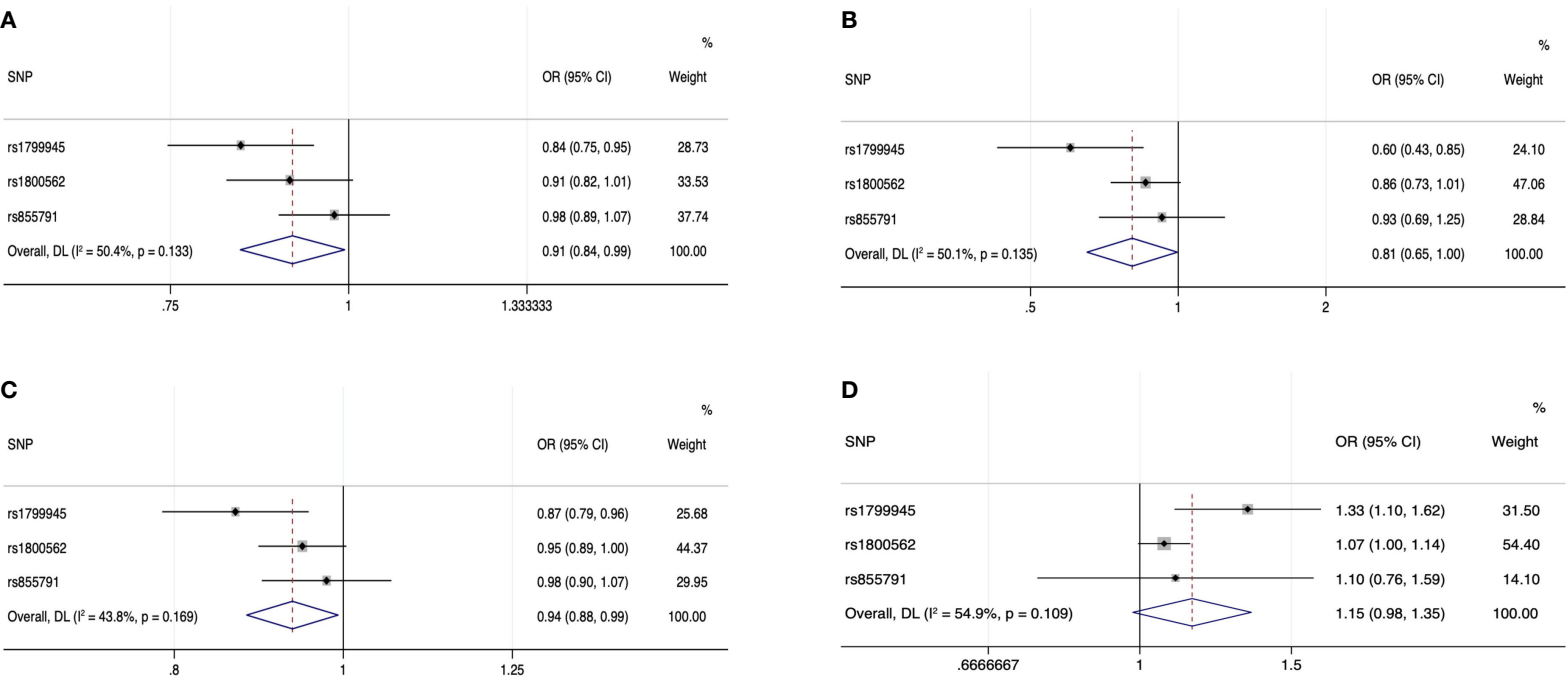
Forest plot of the MR estimates from the three instruments among European populations for serum iron **(A)**, log-transformed ferritin **(B)**, transferrin saturation **(C)**, and transferrin **(D)** on prostate cancer risk. The size of the squares is proportional to the precision of the MR estimates for each SNP, with the horizontal lines indicating their 95% CIs. The combined MR estimate is represented by the center of the diamond, with the lateral tips indicating its 95% CI. The solid vertical line is the line of no effect. Abbreviations: CI, confidence interval; MR, Mendelian randomization; SNP, single-nucleotide polymorphism.

Forest plot of the MR estimates from the three instruments for the four iron biomarkers on the risk of prostate cancer is shown in **Figure 2**. Cochran’s Q test showed that no significant heterogeneity existed among the three instrumental SNPs for the association between iron status and risk of prostate cancer (*P* = 0.133 for serum iron, *P* = 0.135 for log-transformed ferritin, *P* = 0.169 for transferrin saturation, and *P* = 0.109 for transferrin). The effect estimates by IVW method suggested the protective role of increased iron status on prostate cancer, with ORs of 0.91 (95% CI: 0.84, 0.99; *P* = 0.035), 0.81 (95% CI: 0.65, 1.00; *P* = 0.046), 0.94 (95% CI: 0.88, 0.99; *P* = 0.029), and 1.15 (95% CI: 0.98, 1.35; *P* = 0.084) for genetically predicted increased serum iron, log-transformed ferritin, transferrin saturation, and transferrin (**Table 1** **)**. As shown in **Table 1** and **Supplementary Figure 2**, alternative MR methods using simple-median method, weighted-median method, and likelihood-based method produced similar results. The MR-Egger regression did not find potential directional pleiotropy (serum iron: intercept = 0.011, *P* = 0.829; log-transformed ferritin: intercept = −0.006, *P* = 0.805; transferrin saturation: intercept = −0.001, *P* = 0.955; transferrin: intercept = 0.009, *P* = 0.533).

**Table 1:** MR analysis estimates for each iron biomarkers on the risk of prostate cancer among European populations.

Biomarkers and methods	OR (95% CI)	*P*-value for association	*P*-value for Cochran’s Q test	*P*-value for MR-Egger intercept
***Serum iron***				
IVW	0.91 (0.84, 0.99)	0.035	0.133	
MR-Egger	0.87 (0.54, 1.42)	0.585		0.829
Simple median	0.93 (0.86, 0.99)	0.029		
Weighted median	0.91 (0.84, 0.99)	0.024		
Maximum-likelihood	0.92 (0.87, 0.98)	0.005		
***Ferritin (log10)***				
IVW	0.81 (0.65, 1.00)	0.046	0.135	
MR-Egger	0.87 (0.54, 1.40)	0.569		0.805
Simple median	0.86 (0.75, 1.00)	0.048		
Weighted median	0.86 (0.72, 1.02)	0.084		
Maximum-likelihood	0.82 (0.72, 0.94)	0.005		
***Transferrin saturation***				
IVW	0.94 (0.88, 0.99)	0.029	0.169	
MR-Egger	0.94 (0.79, 1.13)	0.521		0.955
Simple median	0.95 (0.90, 1.00)	0.041		
Weighted median	0.95 (0.90, 1.00)	0.047		
Maximum-likelihood	0.94 (0.90, 0.98)	0.004		
***Transferrin***				
IVW	1.15 (0.98, 1.35)	0.084	0.109	
MR-Egger	1.06 (0.91, 1.24)	0.450		0.533
Simple median	1.07 (1.00, 1.15)	0.042		
Weighted median	1.10 (0.92, 1.32)	0.299		
Maximum-likelihood	1.10 (1.03, 1.17)	0.006		

IVW, inverse-variance weighted; MR, Mendelian randomization.

**Figure 3:**
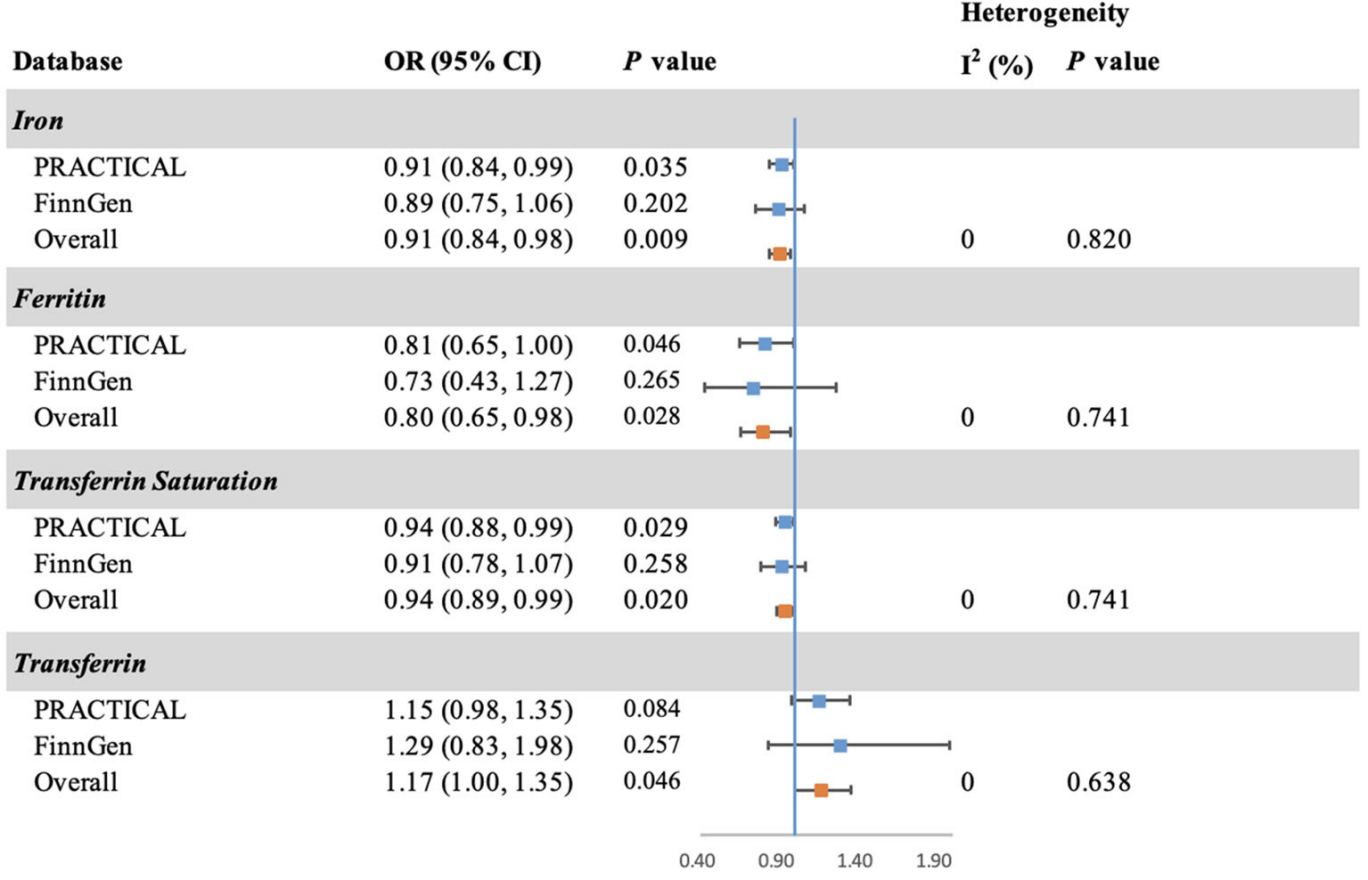
Forest plot of the MR estimates of iron status on prostate cancer in PRACTICAL and FinnGen. The overall odds ratios with 95% CIs were generated using random-effects model. Abbreviations: PRACTICAL, Prostate Cancer Association Group to Investigate Cancer Associated Alterations in the Genome; CI, confidence interval.

The MR analysis was validated using summary statistic generated by FinnGen research project (**Figure 3**). The point estimates in FinnGen gave the compatible directions with our main analysis in PRACTICAL, although the results were not statistically significant. The combined effect sizes were also consistent with our findings in main analysis for iron (OR: 0.91; 95% CI: 0.84, 0.98; *P* = 0.009), ferritin (OR: 0.80; 95% CI: 0.65, 0.98; *P* = 0.028), transferrin saturation (OR: 0.94; 95% CI: 0.89, 0.99; *P* = 0.020), and transferrin (OR: 1.17; 95% CI: 1.00, 1.35; *P* = 0.046).

### 3.2 Meta-analysis

**Supplementary Figure 1** presents the process of study inclusion and exclusion of the meta-analysis. A total of 10 articles (six retrospective studies and four prospective studies) were identified, which investigated the association of serum iron, ferritin, and transferrin saturation with the risk of prostate cancer. We did not perform meta-analysis for transferrin because the number of eligible studies was one. Detailed information of the included studies is displayed in **Table 2**.

**Table 2:** Detailed information about the included studies in the meta-analysis.

Author, year	Study design	Region	No. of cases	No. of all participants	Exposure	NOS
Kuvibidila, 2004	Case-control	America	34	118	Serum iron, ferritin, and transferrin saturation	6
Ozmen, 2006	Case-control	Turkey	20	41	Serum iron	6
Adedapo, 2012	Case-control	Nigeria	40	80	Serum iron	6
Gaur, 2013	Cohort	Sweden	1,505	114,847	Serum iron	8
Kaba, 2014	Case-control	NA	30	62	Serum iron	7
Qayyum, 2014	Case-control	Pakistan	74	140	Serum iron	8
Wen, 2014	Cohort	China	497	141,408	Serum iron	8
Chua, 2016	Cohort	Australia	112	1,597	Serum iron, ferritin, and transferrin saturation	8
Quintana Pacheco, 2018	Case-cohort	Germany	554	1826	Serum iron, ferritin, transferrin saturation, and transferrin	7
Saleh, 2020	Case-control	Saudi Arabia	40	70	Serum iron	7

MR, Mendelian randomization; NA, not available; NOS, Newcastle-Ottawa scale.

Forest plots of the three iron status biomarkers in patients with prostate cancer relative to controls are displayed in **Supplementary Figure 3**. Consistently, we found lower serum ferritin (SMD: −1.25; 95% CI: −2.34, −0.16; *P* = 0.024) and transferrin saturation (SMD: −1.19; 95% CI: –2.34, −0.05; *P* = 0.042) levels in patients with prostate cancer compared with that in controls. However, we did not find statistically significant differences in serum iron levels between cases and controls (SMD: −0.09; 95% CI: −0.35, 0.18; *P* = 0.503). In addition, no evidence of publication bias was detected by Begg’s test (iron: *P* = 0.858, ferritin: *P* = 1.000, transferrin saturation: *P* = 1.000) or Egger’s test (iron: *P* = 0.790, ferritin: *P* = 0.347, transferrin saturation: *P* = 0.396).

## 4 Discussion

In the current study, we performed a MR and meta-analysis to investigate the association of iron status with the risk of prostate cancer. Our results suggested a protective role of iron in the development of prostate cancer.

Several observational studies have found that serum iron biomarkers were higher in patients with prostate cancer than that in the control group. For example, a case-control study of 74 patients with prostate cancer and 66 healthy controls found that the serum iron was significantly higher in patients with prostate cancer compared with that in healthy controls (mean ± standard error: 850.8 ± 46.52 *vs*. 464.3 ± 27.38; *P*< 0.001) (29). Similarly, a recent study conducted by Saleh et al. (30) observed similar result for serum iron (1.96 ± 0.58 *vs*. 1.21 ± 0.27; *P*< 0.001, cases *vs*. controls). On the contrary, a case-control study in America including 34 patients with prostate cancer and 84 controls reported lower serum iron, ferritin, and transferrin saturation in patients with prostate cancer than that in controls (7). Kaba et al. (31) found that the serum iron was lower in the sera samples of 30 patients with prostate cancer than those in 32 healthy controls (0.76 ± 0.43 *vs*. 2.42 ± 0.19; *P*< 0.001). However, the other six studies (two case-control, one case-cohort, and three cohort studies) did not find statistically significant associations between iron status and risk of prostate cancer (8, 32–36). To our knowledge, the current study is the first to systematically review and perform a meta-analysis of the relationship between iron status and the risk of prostate cancer. A total of 260,189 participants (including 2,906 cases) were included in our meta-analysis, which revealed statistically significant associations of increased serum ferritin and transferrin saturation levels with the decreased risk of prostate cancer. However, we did not find associations between serum iron and the risk of prostate cancer, and there was only one study investigating the association of transferrin and prostate cancer, which reported that transferrin was not significantly associated with the risk of prostate cancer.

It is a fact that there were limitations in the meta-analysis. First, because of the limited number of included studies, the result of ferritin and transferrin saturation may not be stable. Second, heterogeneity was at a relatively high level across included observational studies in our meta-analysis. It may result from the study designs, various regions, study quality, baseline characteristics of participants, and potential measurement error. However, stratified analysis was not available for the current study, which needs to be further explored in the future research. Moreover, six of the 10 studies on serum iron were case-control studies with small sample size, which might be biased by reverse causality and confounding. Therefore, the causal direction remains uncertain. Furthermore, we applied a two-sample MR approach to investigate the associations between genetically predicted iron status and the risk of prostate cancer.

To ensure a credible conclusion in the MR study, the selected IVs must satisfy the following three assumptions. The first assumption is that the IV is strongly associated with the exposure. In the current study, all IVs were selected from a GWAS meta-analysis at a genome-wide significance threshold (*P*-value< 5 × 10^−8^). The second assumption is that the IV is not associated with the outcome *via* a confounding pathway. This assumption is like an assurance that all variables except the exposure (which refers to iron status in our study) are distributed equally between the subgroups; hence, no confounders would interfere with the exposure-outcome association. Because genotype is presumed to be randomly allocated at conception, covariates are anticipated to be randomly distributed with respect to genotypes. The third assumption is that the IV affects the outcome through their effects on exposure directly but not through any alternative pathways. Accordingly, we conducted Cochran’s Q test and MR-Egger intercept test, and no potential pleiotropy was detected. Moreover, to prove the stability of the results from MR study, we performed alternative MR methods, i.e., simple-median method, weighted-median method, and likelihood-based method, which gave consistent results on the associations between iron status and the risk of prostate cancer and hence proved the robustness of our findings.

To date, the effect of iron on cancer risk is controversial, and there might be discrepancies in the association of iron with different types of cancer. For example, a cohort study conducted by Ellervik et al. found that participants with higher transferrin saturation levels were more likely to develop liver cancer than those with lower transferrin saturation levels (37). Conversely, a nested case-control study conducted by Fonseca-Nunes et al. found a statistically significant inverse association of ferritin and transferrin with gastric cancer (38). Moreover, rising evidence showed that low systemic iron status and reduced iron intake were associated with the pathogenesis of colorectal cancer (39, 40). Although the mechanism for how serum iron status alters the risk of prostate cancer remains unknown, there are possible hypotheses in support of the protective role of iron in prostate cancer. Iron may protect against prostate cancer through ferroptosis, a form of regulated cell death that is recently found to act in inhibiting some types of cancers including prostate cancer (41). Although ferroptosis is only confirmed to function in pathological cells, a recent study suggested that it may also be triggered in normal cells by accumulation of iron (42). Furthermore, a recent experimental study proved the inhibition of iron toxicity on the proliferation of prostate cancer cells, which primarily affected lipids, promoting ferroptosis (43). Moreover, ferritin is a hollow protein shell composed of two functionally distinct subunits, namely, H-subunit and L-subunit and capable of storing up to 4500 Fe (III) atoms within a single molecule (44). H-subunit acts as a ferroxidase that transforms highly toxic Fe (II) to the mild Fe (III) form, thereby enabling ferritin to sequester it in its cavity (45). Its capability of sequestering free Fe gives ferritin the function of iron detoxification and thus may reduce the incidence of iron overload, causing a decreased risk of prostate cancer. Further investigation is warranted to elucidate the mechanisms behind the protective role of high iron status on prostate cancer.

There are several strengths in our study. First, we used the largest summary statistics of prostate cancer that we know of, which included 79,148 cases and 61,106 controls of European ancestry (13). The large sample size gave us adequate power to estimate the potential causal relationship between iron status and risk of prostate cancer, with 0.87 for serum iron, 0.88 for log-transformed ferritin, 0.89 for transferrin saturation, and 0.86 for transferrin, respectively. Second, our study is the first meta-analysis on the relationship between iron status and risk of prostate cancer, which provides a comprehensive comparison with our MR findings.

There are limitations in our study. First, although the selected IVs reached the genome-wide significant threshold to satisfy the first assumption, the proportion of the variance explained by these selected SNPs for the four biomarkers was limited, with R^2^ of 3.87%, 0.78%, 7.31%, and 3.30% for serum iron, log-transformed ferritin, transferrin saturation, and transferrin, respectively. Therefore, more strong IVs are warranted to improve the power. Second, there can still be potential residual bias as the biological functions of the selected SNPs remains unknown, although our analysis did not find potential pleiotropy of selected SNPs by MR-Egger analysis. Third, because the region of participants was limited to Europe in MR study, whether our finds can be extrapolated to other populations remains unknown. Fourth, our analysis could only detect the linear relation of iron status and risk of prostate cancer due to the limitation of summary-level data used in the MR study. Further analysis with individual-level data is required to evaluate the potential dose-response relationship of iron status and risk of prostate cancer. Finally, our study evaluates the lifelong effect of genetically predicted iron status on the risk of prostate cancer, which does not take the effect of acute changes in iron status into consideration.

In conclusion, our study provided evidence of a potential protective role of iron in the development of prostate cancer. Further studies are warranted to identify the mechanisms, as well as the application prospect of iron supplement in clinical cancer prevention and treatment.

## Data availability statement

Publicly available datasets were analyzed in this study. This data can be found here: PRACTICAL: http://practical.icr.ac.uk, FinnGen: https://finngen.gitbook.io/documentation/.

## Author contributions

DY and XS conceived the idea for the study. SH and JS obtained the genetic data. JY, SH, KL, and WC performed the data analyses. JY, BW, and JS interpreted the results of the data analyses. JY wrote the manuscript, and YM and DY revised the manuscript. All authors contributed to the article and approved the submitted version.
