# Supplementary material for: Genetically predicted iron status was associated with the risk of prostate cancer
Source: Front Oncol. 2022 Dec 6;12:959892. doi: 10.3389/fonc.2022.959892 (PMC9763611; doi:10.3389/fonc.2022.959892)
Supplement: Supplementary file 1 [file DataSheet_1.docx]

**Supplementary Material**

**Supplementary Table 1.** Characteristics of SNPs used for MR analysis among European populations.

**Supplementary Table 2.** Detailed information about the studies and datasets used in our MR study among European populations.

**Supplementary Table 3.** Variances explained by the selected instruments, F-statistics and power calculation for MR analysis among European populations.

**Supplementary Figure 1.** Flowchart of the process of study inclusion and exclusion for meta-analysis.

**Supplementary Figure 2.** Scattered plot of the casual effect of serum iron status on the risk of prostate cancer. The x-axis plots the previously published β-estimate for the association between each SNP and serum phosphorus. The y-axis plots the β-estimate for the association between each SNP and risk of prostate cancer. The lines in different colors indicate the causal effect estimate by inverse variance weighted, maximum likelihood, MR-Egger regression, simple median and weighted median methods. Abbreviations: MR, Mendelian randomization; SNP, single nucleotide polymorphism.

**Supplementary Figure 3.** Forest plot of serum iron (A), serum ferritin (B) and transferrin saturation (C) in patients with prostate cancer relative to controls. The effect sizes (SMDs) with 95% CIs were combined using the random-effects model. The size of the box represents the weight of the study in the combined effect estimate.

Abbreviations: CI, confidence interval; SMD, standardized mean difference.

| **Supplemental Table 1.** Characteristics of SNPs used for MR analysis among European populations^1^. | | | | | | | | | | | | | | | | | | | |  |
| --- | --- | --- | --- | --- | --- | --- | --- | --- | --- | --- | --- | --- | --- | --- | --- | --- | --- | --- | --- | --- |
| SNP | Gene | Effect allele | MAF | Serum iron (μmol/L) | | |  | Log_10_ ferritin (μg/L) | | |  | Transferrin saturation (%) | | | | Transferrin (g/L) | | | |  |
|  |  |  |  | β | SE | *P* |  | β | SE | *P* |  | β | SE | *P* |  | | β | SE | *P* | |
| rs1800562 | *HFE* | A | 0.067 | 0.328 | 0.016 | 2.72×10^-97^ |  | 0.204 | 0.016 | 1.54×10^-38^ |  | 0.577 | 0.016 | 2.19×10^-270^ |  | | -0.479 | 0.016 | 8.90×10^-196^ | |
| rs1799945 | *HFE* | G | 0.150 | 0.189 | 0.010 | 1.10×10^-81^ |  | 0.065 | 0.010 | 1.71×10^-10^ |  | 0.231 | 0.010 | 5.13×10^-100^ |  | | -0.114 | 0.010 | 9.36×10^-30^ | |
| rs855791 | *TMPRSS6* | G | 0.446 | 0.181 | 0.007 | 1.32×10^-139^ |  | 0.055 | 0.007 | 1.38×10^-14^ |  | 0.190 | 0.008 | 6.41×10^-137^ |  | | -0.044 | 0.007 | 1.98×10^-9^ | |
| ^1^ *P* value < 5×10^-8^ for reporting genome-wide significance.  Abbreviations: MAF, minor allele frequency; MR, Mendelian randomization; SE, standard error; SNP, single nucleotide polymorphism. | | | | | | | | | | | | | | | | | | | |  |

| **Supplemental Table 2.** Detailed information about the studies and datasets used in our MR study among European populations. | | | |
| --- | --- | --- | --- |
| **Exposure or outcome** | **Study or consortium** | **Participants** | **Web source** |
| Iron | Benyamin B et al, 2014 [1] | 48,972 individuals of European ancestries | Not available |
| Prostate cancer | Prostate Cancer Association Group to Investigate Cancer Associated Alterations in the Genome (PRACTICAL) consortium [2] | 79,148 prostate cancer cases and 61,106 controls of European ancestries | <http://practical.icr.ac.uk/> |
|  | FinnGen research project [3] | 10,414 prostate cancer cases and 124,994 controls | Introduction:  <https://finngen.gitbook.io/documentation/>  dataset download:  <https://storage.googleapis.com/finngen-public-data-r7/summary_stats/finngen_R7_C3_PROSTATE_EXALLC.gz> |
| Abbreviations: MR, Mendelian randomization. | | | |

| **Supplemental Table 3.** Variances explained by the selected instruments, F-statistics and power calculations for MR analysis among European populations^1^. | | | |
| --- | --- | --- | --- |
| Exposure | Variance explained (R^2^) | F-statistic | Odds ratio estimated for α |
| **Serum iron** | **0.03870** |  | 1.08 or 0.93 |
| rs1800562 | 0.01345 | 667.62 |  |
| rs1799945 | 0.00911 | 450.14 |  |
| rs855791 | 0.01619 | 805.81 |  |
| **Log_10_ ferritin** | **0.00780** |  | 1.19 or 0.84 |
| rs1800562 | 0.00520 | 256.11 |  |
| rs1799945 | 0.00108 | 52.81 |  |
| rs855791 | 0.00150 | 73.31 |  |
| **Transferrin saturation** | **0.07310** |  | 1.06 or 0.94 |
| rs1800562 | 0.04162 | 2126.74 |  |
| rs1799945 | 0.01361 | 675.50 |  |
| rs855791 | 0.01784 | 889.43 |  |
| **Transferrin** | **0.03300** |  | 1.09 or 0.92 |
| rs1800562 | 0.02869 | 1446.14 |  |
| rs1799945 | 0.00331 | 162.82 |  |
| rs855791 | 0.00096 | 46.89 |  |
| ^1^R2, percentage of variations of the iron biomarker explained by the instrumental variables. F-statistic, a function of the magnitude and precision of the genetic effect on the biomarker to evaluate the strength of the instrumental variables. Odds ratio estimated for α, the smallest effect detected by the sample size to provide 80% statistical power at an alpha level of 5%.  Abbreviations: MR, Mendelian randomization. | | | |


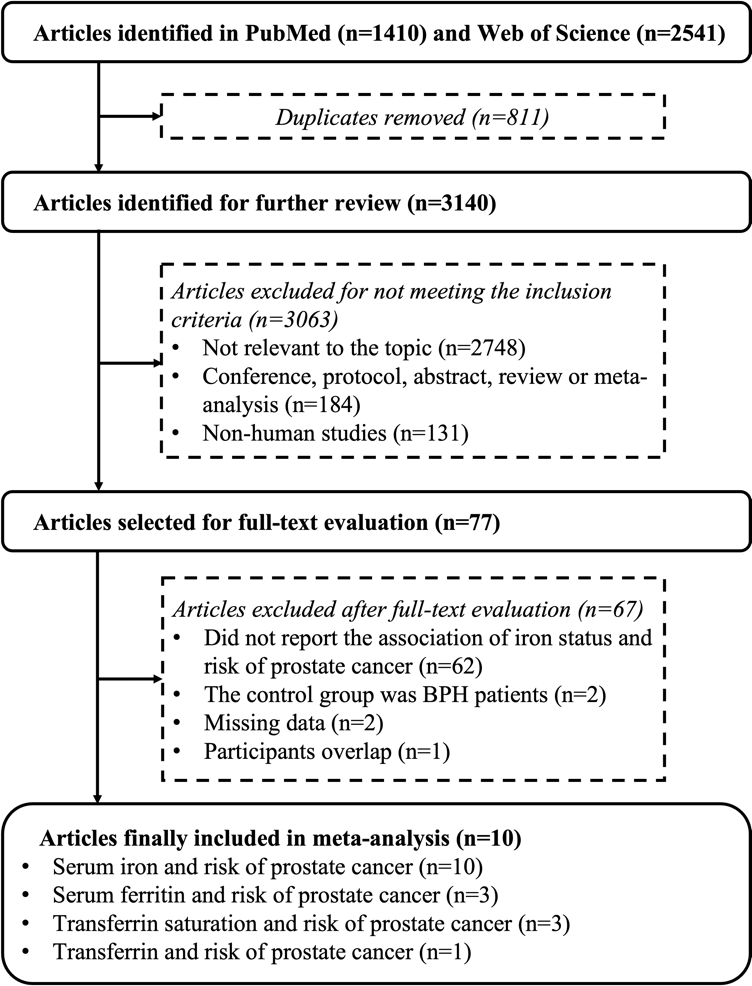


**Supplemental Figure 1.** Flowchart of the process of study inclusion and exclusion for meta-analysis.

**Supplemental Figure 2.** Scattered plot of the casual effect of serum iron status on the risk of prostate cancer. The x-axis plots the previously published β-estimate for the association between each SNP and serum phosphorus. The y-axis plots the β-estimate for the association between each SNP and the risk of prostate cancer. The lines in different colors indicate the causal effect estimate by inverse variance weighted, maximum likelihood, MR-Egger regression, simple median and weighted median methods. Abbreviations: MR, Mendelian randomization; SNP, single nucleotide polymorphism.


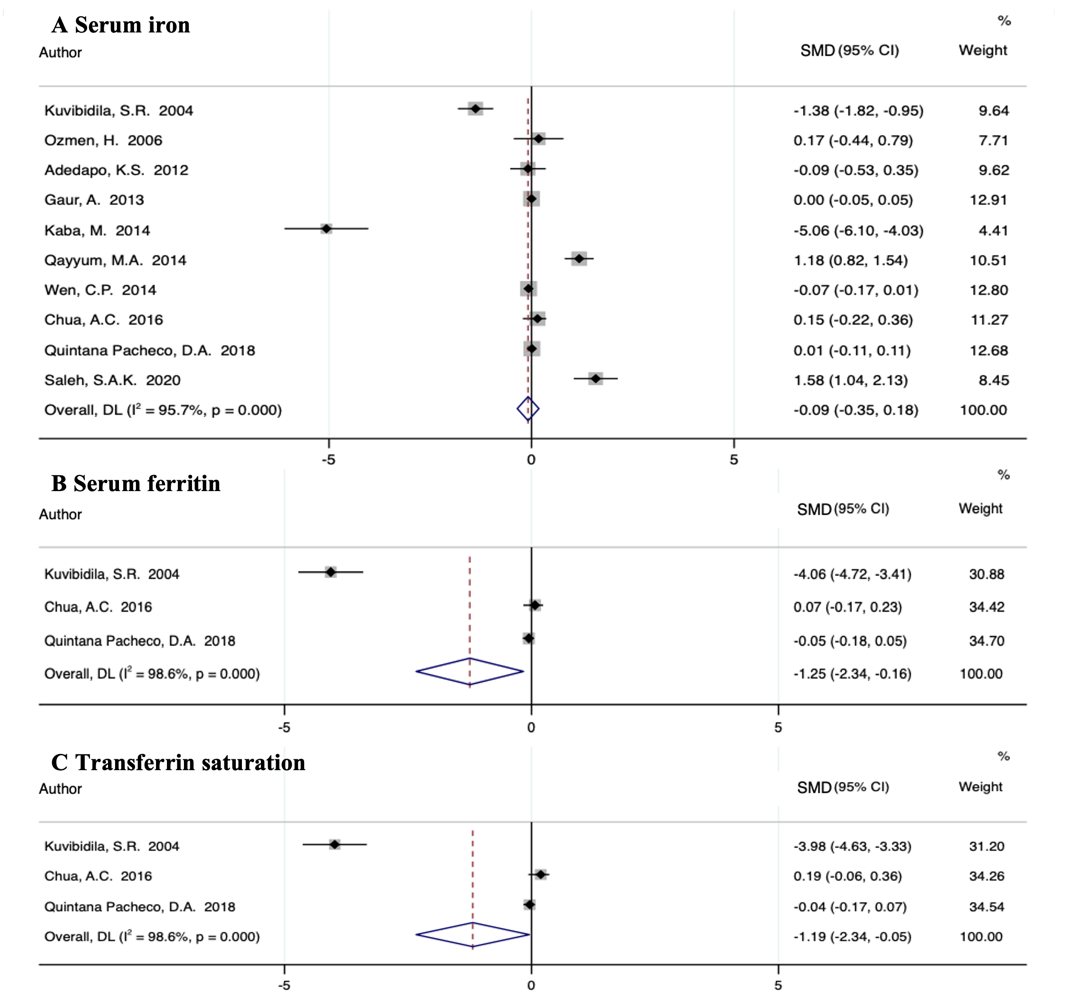


**Supplemental** **Figure 3.** Forest plot of serum iron (A), serum ferritin (B) and transferrin saturation (C) in patients with prostate cancer relative to controls. The effect sizes (SMDs) with 95% CIs were combined using the random-effects model. The size of the box represents the weight of the study in the combined effect estimate.

Abbreviations: CI, confidence interval; SMD, standardized mean difference.

**References**

1. Benyamin B, Esko T, Ried JS, Radhakrishnan A, Vermeulen SH, Traglia M, Gögele M, Anderson D, Broer L, Podmore C *et al*: **Novel loci affecting iron homeostasis and their effects in individuals at risk for hemochromatosis**. *Nat Commun* 2014, **5**:4926.

2. Schumacher FR, Al Olama AA, Berndt SI, Benlloch S, Ahmed M, Saunders EJ, Dadaev T, Leongamornlert D, Anokian E, Cieza-Borrella C *et al*: **Association analyses of more than 140,000 men identify 63 new prostate cancer susceptibility loci**. *Nature genetics* 2018, **50**(7):928-936.

3. Kurki MI, Karjalainen J, Palta P, Sipilä TP, Kristiansson K, Donner K, Reeve MP, Laivuori H, Aavikko M, Kaunisto MA *et al*: **FinnGen: Unique genetic insights from combining isolated population and national health register data**. *medRxiv* 2022:2022.2003.2003.22271360.
